# Uncoupling of glycolysis from glucose oxidation accompanies the development of heart failure with preserved ejection fraction

**DOI:** 10.1186/s10020-018-0005-x

**Published:** 2018-03-15

**Authors:** Natasha Fillmore, Jody L. Levasseur, Arata Fukushima, Cory S. Wagg, Wei Wang, Jason R. B. Dyck, Gary D. Lopaschuk

**Affiliations:** Cardiovascular Research Centre, Mazankowski Alberta Heart Institute University of Alberta, Edmonton, Canada

**Keywords:** Mitochondria, Fatty acid oxidation, Energy metabolism, Diastolic dysfunction, Cardiac hypertrophy

## Abstract

**Background:**

Alterations in cardiac energy metabolism contribute to the development and severity of heart failure (HF). In severe HF, overall mitochondrial oxidative metabolism is significantly decreased resulting in a reduced energy reserve. However, despite the high prevalence of HF with preserved ejection fraction (HFpEF) in our society, it is not clear what changes in cardiac energy metabolism occur in HFpEF, and whether alterations in energy metabolism contribute to the development of contractile dysfunction.

**Methods:**

We directly assessed overall energy metabolism during the development of HFpEF in Dahl salt-sensitive rats fed a high salt diet (HSD) for 3, 6 and 9 weeks.

**Results:**

Over the course of 9 weeks, the HSD caused a progressive decrease in diastolic function (assessed by echocardiography assessment of E’/A’). This was accompanied by a progressive increase in cardiac glycolysis rates (assessed in isolated working hearts obtained at 3, 6, and 9 weeks of HSD). In contrast, the subsequent oxidation of pyruvate from glycolysis (glucose oxidation) was not altered, resulting in an uncoupling of glucose metabolism and a significant increase in proton production. Increased glucose transporter (GLUT)1 expression accompanied this elevation in glycolysis. Decreases in cardiac fatty acid oxidation and overall adenosine triphosphate (ATP) production rates were not observed in early HF, but both significantly decreased as HF progressed to HF with reduced EF (i.e. 9 weeks of HSD).

**Conclusions:**

Overall, we show that increased glycolysis is the earliest energy metabolic change that occurs during HFpEF development. The resultant increased proton production from uncoupling of glycolysis and glucose oxidation may contribute to the development of HFpEF.

**Electronic supplementary material:**

The online version of this article (10.1186/s10020-018-0005-x) contains supplementary material, which is available to authorized users.

## Background

An abundance of evidence indicates that alterations in energy metabolism contribute to the severity of heart failure (Kato et al. 2010; Degens et al. 2006; Lei et al. 2004; Conway et al. 1991; Nascimben et al. 1995; Tian et al. 1996; Beer et al. 2002; Neubauer et al. 1999; Mori et al. 2013). This includes a decrease in overall cardiac mitochondrial oxidative metabolism as the severity of heart failure increases (Conway et al. 1991; Nascimben et al. 1995; Tian et al. 1996; Beer et al. 2002; Neubauer et al. 1999; Lopaschuk et al. 2010). Improving cardiac efficiency, such as by either stimulating glucose oxidation or inhibiting fatty acid oxidation, can help to lessen the impact of this decrease in mitochondrial oxidative capacity, and can improve cardiac function in the failing heart (Kato et al. 2010; Masoud et al. 2014; Yamashita et al. 2009; Ussher et al. 2012; Stanley et al. 2005; Lopaschuk et al. 2003; Dyck et al. 2006; Dyck et al. 2004). However, there is not a consensus as to the importance of the specific energy metabolic changes to the development of heart failure. While it is generally believed that there is an increase in overall glucose metabolism in the failing heart, this may actually be specific to an increase in glucose uptake and glycolysis (Lei et al. 2004; Lopaschuk et al. 2010; Masoud et al. 2014). Whether mitochondrial oxidation of glucose, which supplies the majority of adenosine triphosphate (ATP) derived from glucose, is also increased in heart failure is debatable. In fact, myocardial glucose oxidation rates are decreased in mouse hearts subjected to cardiac hypertrophy and heart failure (Mori et al. 2013; Zhabyeyev et al. 2013; Zhang et al. 2013), and in pig hearts subjected to rapid-pacing induced heart failure (Schroeder et al. 2013).

The specific changes in glycolysis rates and glucose oxidation rates in heart failure are important because uncoupling of glycolysis and glucose oxidation has been shown to impair cardiac function. A selective increase in glycolysis relative to glucose oxidation uncouples glycolysis from glucose oxidation, which can result in the production of lactate and protons (Liu et al. 1996; Liu et al. 2002; Folmes et al. 2006). This rise in protons and drop in pH can reduce contractility of the adult heart by impairing troponin I sensitivity to calcium and inhibiting the slow calcium current (Chesnais et al. 1975; Vogel and Sperelakis 1977; Steenbergen et al. 1977; Schiaffino et al. 1993; Morimoto and Goto 2000). In addition, ATP is utilized to both remove these protons and maintain sodium and calcium homeostasis which decreases cardiac efficiency and contributes to the decrease in cardiac function (Lopaschuk et al. 2010).

Uncoupling of glycolysis and glucose oxidation may also contribute to the development of heart failure by increasing cardiac hypertrophy. Uncoupling of glycolysis and glucose oxidation is present in proliferative cells and is believed to be important in promoting cell growth. Otto Warburg first reported that cancer cells, which are characterized by high rates of proliferation, have high glycolysis rates even under aerobic conditions, a phenomenon called the “Warburg” effect (Vander Heiden et al. 2009; Warburg 1956). In addition, glycolysis is elevated in another form of cell growth, cardiac hypertrophy, which can lead to heart failure (Piao et al. 2010; Piao et al. 2013; Allard et al. 1994; Leong et al. 2002). A similar “Warburg” phenomena may also exist in the failing heart, which is frequently characterized by a relative rise in glycolysis and an overall decrease in mitochondrial oxidative metabolism (Lei et al. 2004; Conway et al. 1991; Nascimben et al. 1995; Tian et al. 1996; Beer et al. 2002; Neubauer et al. 1999; Lopaschuk et al. 2010; Zhang et al. 2013). This suggests that the coupling of glycolysis and glucose oxidation may be a promising target for the treatment of diseases characterized by abnormal cell growth. In fact, stimulation of glucose oxidation (by inhibition of pyruvate dehydrogenase kinase, PDK) has been reported to be beneficial in multiple scenarios, including treatment of cancer, T-cells, cardiac hypertrophy, and heart failure (Kato et al. 2010; Liu et al. 1996; Bonnet et al. 2007; Gerriets et al. 2015).

While there is a substantial amount of evidence to indicate that heart failure with reduced ejection fraction (HFrEF) is commonly characterized by an overall decrease in oxidative metabolism and relative increase in glycolysis (which can result in increased uncoupling of glycolysis and glucose oxidation), there is a scarcity of research on metabolism in another common form of heart failure, heart failure with preserved ejection fraction (HFpEF). In this study we therefore examined mitochondrial oxidative metabolism and glycolysis during the development of HFpEF. This was examined using the Dahl salt-sensitive rat, a well characterized model of HFpEF (Horgan et al. 2014; Rapp and Dene 1985; Klotz et al. 2006), and cardiac energy metabolism was assessed after 3 weeks, 6 weeks, or 9 weeks on a high salt diet (HSD).

## Methods

### Animal protocol

Eight week old male Dahl salt-sensitive rats were either fed a standard low salt diet containing 0.3% NaCl (Research Diets, D10012G) or a high salt diet (HSD) (Research Diets, D11021901) containing 8% NaCl to induce HFpEF. Control rats were kept on the low salt diet while treatment groups were fed the HSD for 3, 6, or 9 weeks. Food and water were provided ad libitum. Rats were kept on a 12 h light:12 h dark cycle. All procedures on animals were approved by the University of Alberta Health Sciences Animal Welfare Committee and conformed to the Canadian Council on Animal Care guidelines (Canadian Council on Animal Care 2017).

### Echocardiography

In vivo cardiac function was assessed in rats anesthetized with 1–1.5% isoflurane using a Vevo 770 high resolution echocardiography imaging system (VisualSonics, Toronto) with a 30-MHz transducer (Zhong et al. 2010). Doppler and tissue doppler imaging were used to assess diastolic function: E’/A’, E’, E/A, E’/E, and isovolumetric relaxation time (IVRT). M-mode images were used to measure % Ejection fraction (%EF) and % Fractional shortening (%FS), to make left ventricle (LV) wall measurements [Interventricular septum end diastole (IVSd), LV internal diameter end diastole (LVIDd), LV posterior wall thickness end diastole (LVPWd), Interventricular septum end systole (IVSs), LV internal diameter end systole (LVIDs), LV posterior wall thickness end systole (LVPWs)], and to measure LV diameter and volume [left ventricular end diastolic diameter, left ventricular end systolic diameter, LV volume end diastole (LV Vol;d), LV volume end systole (LV Vol;s), and corrected LV mass].

### Isolated working heart perfusions

Rats were anesthetized with sodium pentobarbital (1 g/kg BW). Hearts were quickly excised from fully anesthetized rats, and were perfused in the working mode at a 11.5 mmHg left atrial preload and 80 mmHg aortic afterload, as previously described (Liu et al. 1996; Liu et al. 2002). Isolated working hearts were perfused with modified Krebs-Henseleit solution (118.5 mM NaCl, 25 mM NaHCO_3_, 4.7 mM KCl, 1.2 mM MgSO_4_, 1.2 mM KH_2_PO_4_, 2.5 mM CaCl_2_) supplemented with 5 mM glucose, 0.5 mM lactate, and 0.8 mM palmitate bound to 3% fatty acid-free bovine serum albumin (BSA). To measure palmitate oxidation, glucose oxidation, glycolysis, and lactate oxidation [9,10-^3^H] palmitate, [U-^14^C] glucose, [5-^3^H] glucose, or [U-^14^C] lactate, respectively, were added to the Krebs-Henseleit solution. At 30 min of the 60 min perfusion, 100 μU/mL insulin was added to the Krebs-Henseleit solution. Glucose and lactate oxidation rates were assessed by measuring ^14^CO_2_ production. Palmitate oxidation and glycolysis rates were assessed by measuring ^3^H_2_O production. Proton production was determined by subtracting the glucose oxidation rate from the glycolysis rate and multiplying the result by 2 (Liu et al. 2002). Mechanical function was measured using a Powerlab acquisition system and a Transonic flow meter and probes were placed in the preload and afterload lines to measure cardiac output and aortic flow. Cardiac work (joules/min/g dry weight) was calculated by subtracting preload pressure from peak systolic pressure which was then multiplied by cardiac output and normalized against the heart dry weight. At the end of the aerobic perfusion protocol, hearts were immediately frozen in liquid N_2_ and stored at − 80 °C (Barr and Lopaschuk 1997; Ussher et al. 2009).

### Western blot analysis

Standard western blot procedures were followed. Briefly, frozen ventricular tissue was homogenized for 30 s in buffer containing 50 mM Tris HCl, 1 mM EDTA, 10% glycerol, 0.02% Brij-35, 1 mM dithiothreitol (DTT), and protease and phosphatase inhibitors (Sigma). The homogenate was left on ice for 10 min and then centrifuged at 10,000 x g for 20 min. Protein concentration of supernatant was determined using a Bradford protein assay. SDS-polyacrylamide gel electrophoresis was used and protein was transferred onto a 0.45 μm nitrocellulose membrane. Membranes were blocked with 5% fat free milk for 1 h and probed with primary antibodies in 5% BSA overnight. Primary antibodies included pyruvate dehydrogenase (PDH; Cell Signaling 2784), phosphoSer293 PDH (Calbiochem AP1062), phosphoThr389 p70S6K (Cell Signaling 9206), p70S6K (Cell Signaling 9202), phosphoglycerate mutase 1 (PGAM1) (Cell Signaling 7534), hydroxyacyl coenzyme A dehydrogenase (HADH) (Abcam ab93172), GLUT1 (Santa Cruz 1605), GLUT4 (Santa Cruz 1606), lactate dehydrogenase A (LDHA) (Santa Cruz 27,230), mitochondrial pyruvate carrier 1 (MPC1) (Cell Signaling 14,462), MPC2 (Cell Signaling 46,141), cytochrome c (Santa Cruz 8385) and hypoxia inducible factor (HIF)1α (Novus Biologicals 100–105)). Membranes were then washed 4 × 5 min in Tris-buffered saline tween, probed with secondary antibody, goat antirabbit (Santa Cruz 2054), goat antimouse (Santa Cruz 2055), or donkey antigoat (Jackson Immunoresearch 705,035,003), and again washed 4 × 5 min in Tris-buffered saline tween. Protein bands were then visualized with enhanced chemiluminescence (Perkin Elmer). Quantification was performed using Image J.

### Statistical analysis

Values are presented as mean ±SEM. One Way Analysis of Variance (ANOVA) with Bonferroni posthoc test was performed or Kruskal-Wallis test with Dunn’s Multiple Comparison test was performed as appropriate using Prism software to determine statistical significance. Differences are considered significant if p<0.05. n size is indicated in the figure legends.

## Results

### Feeding a high salt diet (HSD) to Dahl salt-sensitive rats results in the progressive development of hypertrophy and diastolic dysfunction

**Fig. 1:**
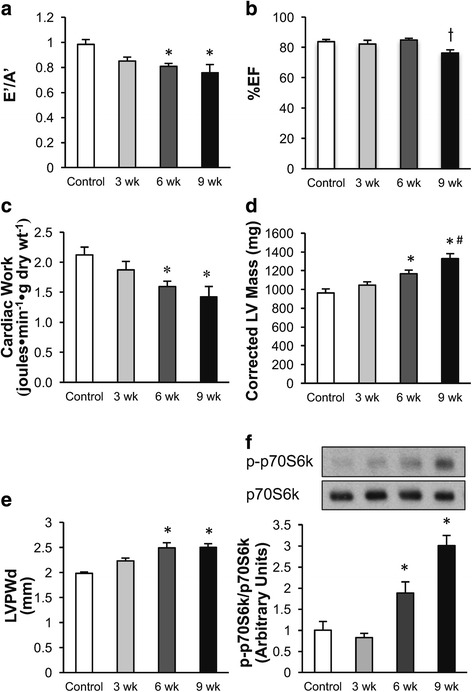
Time dependent effects of a high salt diet (HSD) on Dahl salt-sensitive rat cardiac function and hypertrophy. **a** Diastolic dysfunction was measured by E’/A’. **b** %EF was measured by echocardiography. **c** Cardiac work was measured during the isolated working heart perfusions. **d-e** Cardiac hypertrophy was measured by corrected LV mass and LVPWd using echocardiography. **f** Cardiac p-p70S6K/p70S6K protein expression was assessed in hearts obtained at each of the time periods studied. Measurements were made in Dahl salt-sensitive rats fed a low salt diet, 0.3% NaCl (Control) or a HSD, 8% NaCl, for 3, 6, or 9 weeks. n = 5–8 * *p* < 0.05 compared to Control. # *p* < 0.05 compared to 3 weeks. t *p* < 0.05 compared to 6 weeks. Values shown as mean ± SEM

When Dahl salt-sensitive rats were fed a HSD, a progressive decrease in diastolic function was observed over a 9 week period. In vivo echocardiography of the hearts showed a significant decrease in E’/A’ (a measure of diastolic dysfunction) by 6 weeks following initiation of the HSD (Fig. 1a). In contrast, systolic function was largely preserved, with %EF decreasing slightly by 9 weeks (Fig. 1b), and %FS remaining unchanged (Table 1). This suggests that the rats developed HFpEF prior to developing heart failure with reduced ejection fraction (HFrEF). In isolated working hearts obtained from rats at each time period studied a progressive decrease in cardiac work was observed over the 9 week period (Fig. 1c), primarily due to a decrease in cardiac output (Table 2). This time frame for heart failure development agrees with other studies examining the development of heart failure in DSS rats fed a HSD (Rapp and Dene 1985; Klotz et al. 2006). The progressive development of diastolic dysfunction in the Dahl salt-sensitive rats following administration of the HSD was accompanied by an increase in LV mass (Fig. 1d), as well as an increase in IVSd and LVPWd (Fig. 1e, Table 1). At the same time the phosphorylation of p70S6k increased suggesting a stimulation of the mTOR pathway (which promotes cardiac hypertrophy) (Fig. 1f).

**Table 1:** Effect of a high salt diet (HSD) on in vivo cardiac function in Dahl salt-sensitive rats

	Control	3 weeks	6 weeks	9 weeks
EF (%)	83.79 ±1.44	82.21 ±2.50	84.91 ±1.14	76.21 ±2.19^T^
FS (%)	54.60 ±1.69	53.12 ±2.57	54.45 ±2.39	47.64 ±2.34
LV Mass Corrected	962.88 ±43.45	1045.85 ±36.11	1167.85 ±38.96^*^	1328.94 ±52.37^*#^
IVSd (mm)	1.97 ±0.05	2.23 ±0.06^*^	2.40 ±0.07^*^	2.50 ±0.07^*#^
LVIDd (mm)	7.91 ±0.12	7.32 ±0.14^*^	7.45 ±0.09	7.52 ±0.20
LVPWd (mm)	1.98 ±0.03	2.23 ±0.06	2.49 ±0.10^*^	2.50 ±0.08^*^
IVSs (mm)	3.40 ±0.05	3.67 ±0.10	3.68 ±0.09	3.68 ±0.15
LVIDs (mm)	3.87 ±0.18	3.46 ±0.23	3.40 ±0.09	4.25 ±0.08
LVPWs (mm)	3.45 ±0.12	3.68 ±0.11	3.77 ±0.08	3.65 ±0.13
LV Vol;d	335.75 ±10.95	283.11 ±12.35^*^	294.62 ±7.78	301.74 ±17.16
LV Vol;s	51.99 ±6.28	45.76 ±6.96	47.51 ±3.37	81.30 ±3.62^*#T^
E/E’	19.04 ±2.18	20.49 ±1.95	18.82 ±1.22	18.29 ±1.51
E/A	1.60 ±0.11	1.64 ±0.07	1.33 ±0.15	1.40 ±0.20
E’/A’	0.98 ±0.04	0.85 ±0.03	0.81 ±0.02^*^	0.76 ±0.07^*^
Tei Index	0.65 ±0.03	0.72 ±0.04	0.78 ±0.05	0.76 ±0.05
E’	54.23 ±6.85	54.68 ±4.61	46.79 ±2.14	38.26 ±3.49
IVRT (ms)	24.32 ±0.60	22.68 ±0.68	27.97 ±1.76	28.13 ±1.63
IVCT (ms)	15.69 ±0.90	16.88 ±1.29	15.81 ±1.48	17.47 ±1.43
HR (bpm)	365.25 ±6.61	375.88 ±7.71	367.63 ±10.98	349.75 ±16.68
L Kidney/TL (g/cm)	0.31 ±0.01	0.34 ±0.01	0.34 ±0.01	0.38 ±0.03^*^

In vivo cardiac function was measured via echocardiography in Dahl salt-sensitive rats fed a low salt diet, 0.3% NaCl (Control), or a high salt diet, 8% NaCl, for 3, 6, or 9 weeks. *n* = 5–8 * *p* < 0.05 compared to Control. * *p* < 0.05 compared to Control. ^#^ *p* < 0.05 compared to 3 weeks. ^T^ *p* < 0.05 compared to 6 weeks. *n* = 6–9 Values shown as mean ±SEM % Ejection fraction (%EF); % Fractional shortening (%FS); left ventricle (LV); Interventricular septum end diastole (IVSd); LV internal diameter end diastole (LVIDd); LV posterior wall thickness end diastole (LVPWd); Interventricular septum end systole (IVSs); LV internal diameter end systole (LVIDs); LV posterior wall thickness end systole (LVPWs); LV volume end diastole (LV Vol;d); LV volume end systole (LV Vol;s); Isovolumetic relaxation time (IVRT); Isovolumetic contraction time (IVCT); Tibia length (TL); Heart rate (HR)

**Table 2:** Effect of a high salt diet (HSD) on cardiac function ex vivo in Dahl salt-sensitive rats

	Control	3 weeks	6 weeks	9 weeks
Heart rate (beats●min^− 1^)	277.9 ±7.3	290.8 ±8.6	278.7 ±6.1	266.9 ±22.9
Peak Systolic Pressure (mmHg)	109.9 ±1.0	107.8 ±2.6	110.8 ±1.7	107.5 ±6.7
Developed Pressure (mmHg)	41.9 ±2.8	38.5 ±3.8	42.6 ±2.2	43.1 ±7.0
Cardiac Output (ml●min^−1^)	50.4 ±2.2	45.7 ±1.9	41.6 ±2.2	38.5 ±3.1^*^
Coronary Flow (ml●min^−1^)	26.5 ±2.8	23.9 ±0.5	22.3 ±0.7	20.8 ±1.5
Cardiac Work (joules●min^− 1^●g dry weight^− 1^)	2.1 ±0.1	1.9 ±0.1	1.6 ±0.1^*^	1.4 ±0.2^*^

Cardiac function was measured in isolated working hearts from Dahl salt-sensitive rats fed a low salt diet, 0.3% NaCl (Control), or a high salt diet, 8% NaCl, for 3, 6, or 9 weeks. *n* = 3–5 * *p* < 0.05 compared to Control. Values shown as mean ±SEM

### The development of diastolic dysfunction is accompanied by a decrease in cardiac mitochondrial oxidative metabolism in dahl salt-sensitive rats

**Fig. 2:**
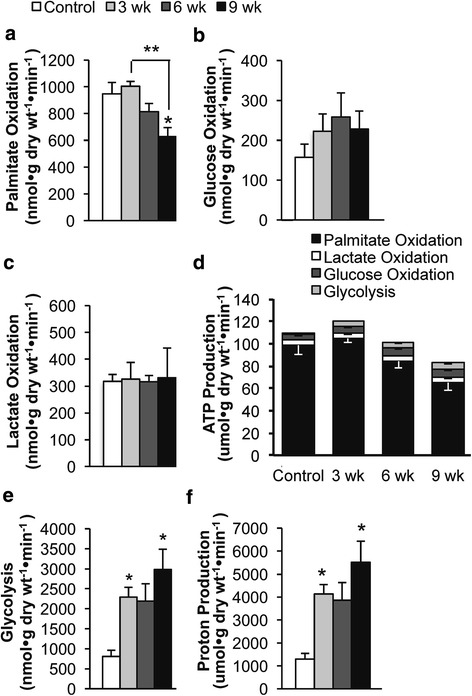
Time dependent effects of a high salt diet (HSD) on palmitate oxidation, glucose oxidation, glycolysis, and lactate oxidation in Dahl salt-sensitive rat hearts. **a** Palmitate oxidation, (**b**) Glucose oxidation, (**c**) Lactate oxidation, (**d**) ATP production, (**e**) Glycolysis, and (**f**) Proton production were measured in hearts from Dahl salt-sensitive rats fed a low salt diet, 0.3% NaCl (Control) or a HSD, 8% NaCl, for 3, 6, or 9 weeks. Energy metabolic rates were assessed in isolated working hearts. Proton production was calculated based on glycolysis and glucose oxidation rates. Contribution to ATP production was calculated from the metabolic rates assessed using the isolated working heart perfusion in Dahl salt-sensitive rats. *n* = 3–5 * *p* < 0.05 compared to Control. Values shown as mean ± SEM

Rates of overall energy metabolism were measured in isolated working hearts obtained at 3 weeks, 6 weeks, and 9 weeks of the HSD. A progressive decrease in fatty acid oxidation rates was observed following the HSD (Fig. 2a), although a significant decrease in fatty acid oxidation rates did not occur until 9 weeks of the HSD. In the presence of insulin, a similar time-dependent decrease in fatty acid oxidation was also observed (Table 3). In contrast, there was no change in glucose oxidation rates (Fig. 2b) or lactate oxidation rates (Fig. 2c) during the development of diastolic dysfunction, regardless of whether insulin was present (Table 3) or absent. The primary source of overall cardiac ATP production in all hearts originated from fatty acid oxidation (Fig. 2d). As a result, a decrease in overall cardiac ATP production rates were observed by 9 weeks of the HSD, which was primarily due to the observed decrease in fatty acid oxidation rates (Fig. 2d).

**Table 3:** Effect of insulin (100 μU/ml) on cardiac metabolism in Dahl salt-sensitive rats fed a high salt diet (HSD)

	Control	3 weeks	6 weeks	9 weeks
Glycolysis	1231.3 ±234.8	2390.9 ±390.4	2090.6 ±647.2	2982.2 ±734.1
Glucose Oxidation	312.2 ±20.6	519.0 ±119.7	464.8 ±113.6	493.0 ±166.7
Palmitate Oxidation	983.5 ±47.5	931.0 ±38.2	796.9 ±43.8	616.3 ±56.9^*^
Lactate Oxidation	377.0 ±46.2	426.5 ±95.0	339.6 ±62.4	275.0 ±113.7
Proton Production	1838.3 ±454.1	3743.8 ±558.6	3251.6 ±1073.8	4978.3 ±1197.6
ATP Production				
Glycolysis	2.5 ±0.5	4.8 ±0.8	4.2 ±1.3	6.0 ±1.5
Glucose Oxidation	9.1 ±0.6	15.1 ±3.5	13.5 ±3.3	14.3 ±4.8
Palmitate Oxidation	102.3 ±4.9	96.8 ±4.0	82.9 ±4.6	64.1 ±5.9^*#^
Lactate Oxidation	5.5 ±0.7	6.2 ±1.4	4.9 ±0.9	4.0 ±1.6
Total	115.6 ±3.3	122.8 ±6.2	106.4 ±10.2	88.3 ±11.0

Energy metabolic rates (nmol●g dry wt^−1^●min^−1^) were measured during the working heart perfusion. Contribution to ATP production (μmol●g dry wt^− 1^●min^− 1^) was calculated from the metabolic rates assessed via the isolated working heart perfusion in Dahl salt-sensitive rats. These results are from Dahl salt-sensitive rats fed a low salt diet, 0.3% NaCl (Control), or a high salt diet, 8% NaCl, for 3, 6, or 9 weeks. n = 3–5 * *p* < 0.05 compared to Control. # *p* < 0.05 compared to 3 weeks. Values shown as mean ±SEM

### Decreased glycolysis is an early energy metabolic change in hearts from Dahl salt-sensitive rats fed a HSD

The earliest energy metabolic change that occurred in Dahl-sensitive rats fed a HSD was an increase in glycolysis, which had already increased over 300% by 3 weeks of the HSD (Fig. 2e). This increase was also observed when insulin was present in the perfusate (Table 3), and prior to the onset of either HFpEF or HFrEF (Fig. 1). Since the increase in glycolysis during the development of diastolic dysfunction was not accompanied by an increase in glucose oxidation (Fig. 2b), an uncoupling of glycolysis from glucose oxidation occurred, resulting in a significant increase in proton production, even by 3 weeks of the HSD (Fig. 2f). This increased uncoupling of glycolysis and glucose oxidation and rise in proton production persisted in hearts perfused in the presence of insulin (Table 3). Since the uncoupling of glycolysis and glucose oxidation and elevation in proton production occurs early during the development of diastolic dysfunction, a possible causal link between the uncoupling of glycolysis and glucose oxidation and the development of HFpEF may exist.

Even though glycolysis rates in the hearts remained elevated as HFrEF developed (i.e. by 9 weeks of the HSD), the increase in ATP production originating from glycolysis did not compensate for the decrease in ATP production that occurred as a result of the decrease in fatty acid oxidation (Fig. 2d, Table 3).

### Increased GLUT1 expression may contribute to the increased uncoupling of glycolysis from glucose oxidation observed during the development of diastolic dysfunction

**Fig. 3:**
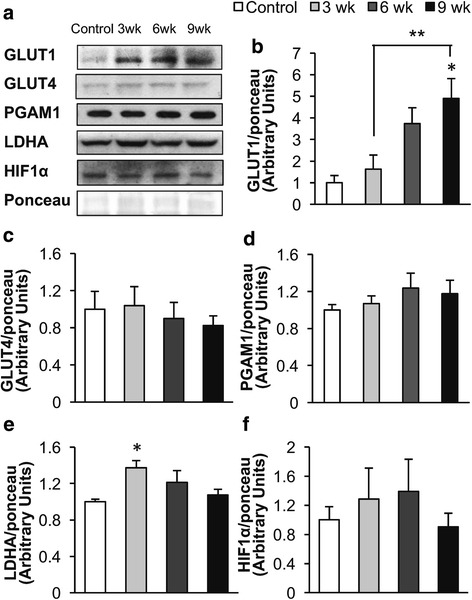
Effect of a high salt diet (HSD) on Dahl salt-sensitive rat heart glucose metabolic enzymes. **a** Representative western blots. **b** GLUT1, (**c**) GLUT4, (**d**) PGAM1, (**e**) LDHA expression, and (**f**) HIF1α protein expression was measured in hearts from Dahl salt-sensitive rats fed either a low salt diet, 0.3% NaCl (Control) or a HSD, 8% NaCl, for 3, 6, or 9 weeks. *n* = 4–9 * *p* < 0.05 compared to Control. ** *p* < 0.05 between compared groups. Values shown as mean ± SEM

Examination of the expression of various proteins involved in glucose metabolism indicated that a change in glucose transport may contribute to the rise in glycolysis seen during the development of diastolic dysfunction. A progressive increase in GLUT1 expression was seen in Dahl salt-sensitive rat hearts during the HSD (Fig. 3a and b). Since GLUT1 mediates glucose uptake independent of insulin, it suggests that increased GLUT1 expression may be involved in the elevated glycolysis rates observed even when hearts were perfused without insulin (Fig. 2e). While PGAM1 and GLUT4 (the insulin-dependent glucose transporter) expression were not significantly altered during the development of diastolic dysfunction, LDHA was significantly increased after 3 weeks on the HSD (Fig. 3d). This isoform of LDH favors the conversion of pyruvate to lactate. This suggests that LDHA may contribute to the initial increase in uncoupling of glycolysis and glucose oxidation observed in response to the HSD. HIF1α, a transcription factor regulating glycolysis, was not altered by the HSD (Fig. 3e).

**Fig. 4:**
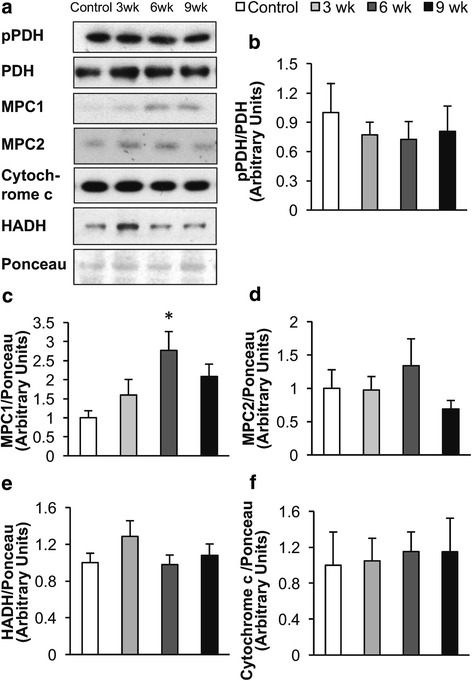
Effect of a high salt diet (HSD) on the cardiac expression of proteins involved in oxidative metabolism. **a** Representative western blots. **b** pPDH Ser293/PDH, (**c**) MPC1, (**d**) MPC2, (**e**) HADH, and (**f**) cytochrome c protein expression was measured in hearts from Dahl salt-sensitive rats fed a low salt diet, 0.3% NaCl (Control) or a HSD, 8% NaCl, for 3, 6, or 9 weeks. *n* = 4–9 * *p* < 0.05 compared to Control. Values shown as mean ± SEM

We also examined the expression of mitochondrial enzymes that might contribute to the changes in cardiac energy metabolism observed in response to the HSD. No significant changes were observed in PDH expression, the rate-limiting enzyme for glucose oxidation (Fig. 4a). While phosphorylation of PDH by PDH kinase decreases PDH activity, we observed no change in pPDH during the development of diastolic dysfunction (Fig. 4a and b). This lack of change in pPDH correlates with the lack of change in glucose oxidation rates during the development of diastolic dysfunction (Fig. 2b). We also looked at the expression of the mitochondrial pyruvate carrier. Interestingly, MPC1 expression was increased after 6 weeks on the HSD, but MPC2 expression was not significantly altered (Fig. 4c and d). In addition, cytochrome c protein expression was not significantly altered (Fig. 4f). Since acetylation has been shown to regulate mitochondrial oxidative metabolism we also assessed the effect of the HSD on overall acetylation. However, overall lysine acetylation was not significantly altered in hearts of Dahl salt-sensitive rats fed a HSD (Additional file 1).

## Discussion

Alterations in cardiac energy metabolism are thought to be an important contributor to the severity of heart failure. However, there is confusion as to what changes in cardiac energy metabolism occur in heart failure, although it is generally believed that in HFrEF fatty acid metabolism decreases while overall glucose metabolism increases (Kato et al. 2010; Ingwall 2007; Davila-Roman et al. 2002). In this study we directly determined for the first time that the earliest cardiac energy metabolic changes that occurs in HFpEF is a dramatic increase in glycolysis. This metabolic change occurs prior to the development of HFpEF. Of interest, is that this increase in glycolysis occurs without a parallel change in glucose oxidation, which results in increased uncoupling of glycolysis and glucose oxidation. This is notable as previously published research has shown that increasing this uncoupling of glycolysis and glucose oxidation can result in intracellular acidosis, which can impair cardiac function (Liu et al. 1996; Liu et al. 2002; Steenbergen et al. 1977). An increased uncoupling of glycolysis and glucose oxidation has also been reported in other more severe models of heart failure such as in rodent hearts subjected to coronary artery ligation (CAL) (Masoud et al. 2014). Further, in one study abdominal aortic banding induced changes in cardiac metabolite levels indicative of increased uncoupling of glycolysis and glucose oxidation due to elevated glycolysis (Seymour et al. 2015). Our data also confirms that fatty acid oxidation decreases in HFrEF. There is evidence indicating that a reduction in fatty acid oxidation may contribute to diastolic dysfunction. For example, it was recently reported that overexpressing Acetyl Coenzyme A Carboxylase both prevented diastolic dysfunction and reduced cardiac fatty acid oxidation in mice treated with Angiotensin II (Choi et al. 2016; Helge et al. 1996). However, of importance is that the decrease in fatty acid oxidation seen in the failing heart in our study did not precede the onset of diastolic dysfunction. As a result, it is unlikely that a decrease in fatty acid oxidation is contributing to the early development of diastolic dysfunction in heart failure.

While it is often cited that in heart failure the heart switches from fatty acid to glucose metabolism, our data suggests that it is more accurate to suggest that a decrease in overall cardiac mitochondrial oxidative metabolism occurs in HFrEF, accompanied by a relative increase in glycolysis. Despite glucose oxidation being the major source of glucose derived ATP production, glucose oxidation rates are not increased during the development of systolic dysfunction (Kato et al. 2010; Lopaschuk et al. 2010; Zhabyeyev et al. 2013; Zhang et al. 2013; Liu et al. 1996; Liu et al. 2002). In fact, in mouse models of HFrEF, we actually observed a decrease in glucose oxidation rates (Zhabyeyev et al. 2013; Zhang et al. 2013).

There are several potential explanations for this rise in cardiac glycolysis in diastolic dysfunction. One possibility may be that the overall decrease in mitochondrial oxidative metabolism (which we have previously reported in hearts from HFpEF mice), results in a compensatory rise in glycolysis (Kato et al. 2010; Beer et al. 2002; Masoud et al. 2014; Zhang et al. 2013; Neubauer 2007). The decrease in cardiac ATP production after 9 weeks on a HSD appears to be solely due to a drop in fatty acid oxidation, as glucose and lactate oxidation remained unchanged. In an effort to determine what was responsible for this decrease in fatty acid oxidation we examined the expression of enzymes involved in fatty acid oxidation and mitochondrial oxidative metabolism. It has been previously reported that the cardiac expression of proteins involved in fatty acid oxidation and overall mitochondrial oxidative metabolism are decreased in severe heart failure (Zhang et al. 2013; Lai et al. 2014). However, we did not observe a change in the expression of ß-hydroxyacyl CoA dehydrogenase or cytochrome c in the heart (Fig. 4), enzymes involved in fatty acid oxidation and mitochondrial oxidative metabolism, respectively. Alternatively, the decrease in cardiac work may be lowering cardiac fatty acid oxidation rates. Cardiac fatty acid oxidation rates remained unchanged during the development of diastolic dysfunction when normalized against cardiac work (Control, 410.4 ±41.8; 3 weeks, 498.5 ±38; 6 weeks, 502.2 ±31.4; 9 weeks, 388.0 ±16.1 nmol●g dry wt^− 1^●min^− 1^) (Online Resource 1). While it is possible that a reduction in fatty acid oxidation is causing the decrease in cardiac function, we speculate that the reduction in cardiac fatty acid oxidation is a consequence of reduced cardiac work.

Increased capacity for glycolysis or glucose transport may also be responsible for the rise in glycolysis during development of diastolic dysfunction and heart failure. Although we did not observe a change in the expression of the glycolytic enzyme PGAM1, we did find an increase in LDHA protein expression after 3 weeks on the HSD and a later rise in GLUT1 protein expression (Fig. 4). LDH isoforms that contain LDHA are more likely to convert pyruvate to lactate, as opposed to catalyzing the opposite reaction. Therefore, increased LDHA protein expression could be contributing to the early rise in glycolysis and increased uncoupling of glycolysis and glucose oxidation in response to the HSD.

Increased cardiac GLUT1 expression may also contribute to the rise in glycolysis. In support of this, glycolysis is elevated in hearts overexpressing GLUT1 and is decreased in hearts lacking GLUT1 (Liao et al. 2002; Pereira et al. 2014). However, studies that regulate GLUT1 expression report mixed results on the role of GLUT1 in the development of heart failure. While overexpression of GLUT1 has been reported to prevent pressure overload induced heart failure in mouse hearts, deletion of GLUT1 does not affect the rate of development of pressure overload induced heart failure. The GLUT1 knockout mouse has elevated fatty acid oxidation and reduced glucose oxidation, which would be expected to decrease cardiac efficiency and may explain why these hearts are not resistant pressure overload induced heart failure (Pereira et al. 2014). However, the results from these two studies do not preclude the possibility that a more acute up-regulation of GLUT1 expression could increase glycolysis and impair cardiac function.

Based on these results we hypothesize that stimulating glucose oxidation may be a promising strategy for treating and potentially preventing the development of HFpEF. As mentioned earlier, stimulating cardiac glucose oxidation is associated with an increase in cardiac efficiency and an improvement in cardiac function (Kato et al. 2010; Masoud et al. 2014; Yamashita et al. 2009; Ussher et al. 2012; Stanley et al. 2005; Lopaschuk et al. 2003; Dyck et al. 2006; Dyck et al. 2004). Stimulating cardiac glucose oxidation can also be beneficial in the context of obesity and diabetes, which can lead to heart failure (Ussher et al. 2009; Lewis et al. 2016; Nicholl et al. 1991). Furthermore, treatment of Dahl salt sensitive rats with dichloroacetate (DCA) decreases plasma lactate levels (an indirect indication of elevated glycolysis), and improves cardiac function (Kato et al. 2010). However, in this study intervention with DCA was at a later stage of heart failure development, which was also associated with changes in systolic function. In the future it will be important to determine if stimulating glucose oxidation with more potent PDK inhibitors can lessen or even prevent the development of HFpEF.

## Conclusions

This study directly characterized the changes in cardiac energy metabolism that occur in diastolic dysfunction. We demonstrate that the earliest cardiac metabolic change that occurs during the development of diastolic dysfunction is an increase in glycolysis, with no change in carbohydrate or fatty acid oxidation. The rise in glycolysis resulted in increased uncoupling of glycolysis and glucose oxidation and an increased proton production, which occurs early during the development of diastolic dysfunction. Our findings combined with previous work suggest that the coupling of glycolysis and glucose oxidation is important in maintaining normal cardiac function and may contribute to the development of HFpEF. While these results suggest that decreasing the uncoupling of glycolysis and glucose oxidation may be a promising strategy for the treatment of heart failure, more work is needed to determine if therapeutically improving the coupling of glycolysis and glucose oxidation can treat HFpEF.

## Additional file

Additional file 1: **Figure S1.** Effect of a high salt diet (HSD) on overall protein acetylation. Total protein acetylation levels were measured in hearts from Dahl salt-sensitive rats fed a low salt diet, 0.3% NaCl (Control) or a HSD, 8% NaCl, for 3, 6, or 9 wk. *n* = 6–9 Values shown as mean ± SEM. (TIFF 3225 kb)

## Abbreviations

%EF: % Ejection fraction
%FS: % Fractional shortening
ANOVA: One Way Analysis of Variance
ATP: Adenosine triphosphate
Bpm: beats per minute
BSA: Bovine serum albumin
CAL: Coronary artery ligation
DCA: Dichloroacetate
DTT: Dithiothreitol
GLUT1: Glucose transporter 1
GLUT4: Glucose transporter 4
HADH: Hydroxyacyl coenzyme A dehydrogenase
HF: Heart failure
HFpEF: Heart failure with preserved ejection fraction
HFrEF: Heart failure with reduced ejection fraction
HIF1α: Hypoxia inducible factor 1α
HR: Heart rate
HSD: High salt diet
IVRT: Isovolumetric relaxation time
IVSd: Interventricular septum end diastole
IVSs: Interventricular septum end systole
LDHA: Lactate dehydrogenase A
LV Vol;d: Left ventricle volume end diastole
LV Vol;s: Left ventricle volume end systole
LV: Left ventricle
LVIDd: Left ventricle internal diameter end diastole
LVIDs: Left ventricle internal diameter end systole
LVPWd: Left ventricle posterior wall thickness end diastole
LVPWs: Left ventricle posterior wall thickness end systole
MPC1: Mitochondrial pyruvate carrier 1
MPC2: Mitochondrial pyruvate carrier 2
PDH: Pyruvate dehydrogenase
PGAM1: Phosphoglycerate mutase 1
