# Supplementary figures and images for: Uncoupling of glycolysis from glucose oxidation accompanies the development of heart failure with preserved ejection fraction
Source: Mol Med. 2018 Mar 15;24:3. doi: 10.1186/s10020-018-0005-x (PMC6016884; doi:10.1186/s10020-018-0005-x)

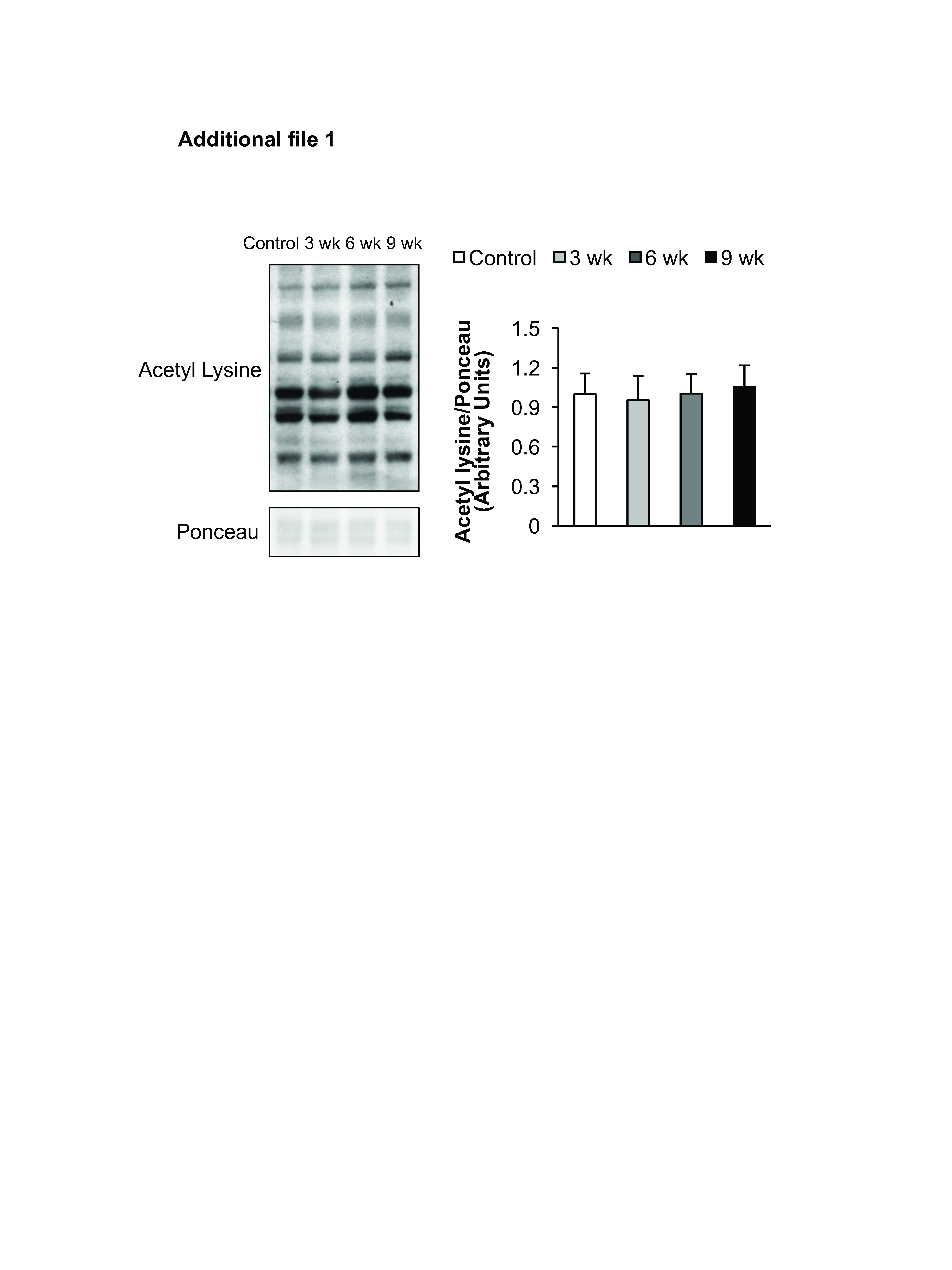

Supplement: Supplementary file 1 — Figure S1. Effect of a high salt diet (HSD) on overall protein acetylation. Total protein acetylation levels were measured in hearts from Dahl salt-sensitive rats fed a low salt diet, 0.3% NaCl (Control) or a HSD, 8% NaCl, for 3, 6, or 9 wk. n = 6–9 Values shown as mean ± SEM. (TIFF 3225 kb) [file 10020_2018_5_MOESM1_ESM.tiff]
